# Cost-Effectiveness of Preemptive Switching to Efavirenz-Based Antiretroviral Therapy for Children With Human Immunodeficiency Virus

**DOI:** 10.1093/ofid/ofz276

**Published:** 2019-06-11

**Authors:** Sophie Desmonde, Simone C Frank, Ashraf Coovadia, Désiré L Dahourou, Taige Hou, Elaine J Abrams, Madeleine Amorissani-Folquet, Rochelle P Walensky, Renate Strehlau, Martina Penazzato, Kenneth A Freedberg, Louise Kuhn, Valeriane Leroy, Andrea L Ciaranello

**Affiliations:** 1 UMR 1027 Inserm, Université Paul Sabatier, Toulouse; 2 Bordeaux School of Public Health, France; 3 Medical Practice Evaluation Center, Boston; 4 Division of General Internal Medicine, Department of Medicine, Boston; 5 Division of Infectious Diseases, Department of Medicine, Massachusetts General Hospital, Boston; 6 Center for AIDS Research, Harvard University, Boston; 7 Department of Health Policy and Management, Harvard T. H. Chan School of Public Health, Boston; 8 Harvard Medical School, Boston; 9 ICAP at Columbia University, Mailman School of Public Health, and Vagelos College of Physicians & Surgeons, Columbia University, New York; 10 Gertrude H. Sergievsky Center, College of Physicians and Surgeons and Department of Epidemiology, Mailman School of Public Health, Columbia University Medical Center, New York, New York; 11 Empilweni Service and Research Unit, Johannesburg, South Africa; 12 Centre Muraz, Bobo-Dioulasso, Burkina Faso; 13 Division of Pediatrics, University Hospital of Cocody, Abidjan, Cote d’Ivoire; 14 World Health Organization, Geneva, Switzerland

**Keywords:** Africa, cost-effectiveness, HIV, pediatrics, treatment strategies

## Abstract

**Background:**

The NEVEREST-3 (South Africa) and MONOD-ANRS-12206 (Côte d’Ivoire, Burkina Faso) randomized trials found that switching to efavirenz (EFV) in human immunodeficiency virus–infected children >3 years old who were virologically suppressed by ritonavir-boosted lopinavir (LPV/r) was noninferior to continuing o LPV/r. We evaluated the cost-effectiveness of this strategy using the Cost-Effectiveness of Preventing AIDS Complications–Pediatric model.

**Methods:**

We examined 3 strategies in South African children aged ≥3 years who were virologically suppressed by LPV/r: (1) continued LPV/r, even in case of virologic failure, without second-line regimens; continued on LPV/r with second-line option after observed virologic failure; and preemptive switch to EFV-based antiretroviral therapy (ART), with return to LPV/r after observed virologic failure. We derived data on 24-week suppression (<1000 copies/mL) after a switch to EFV (98.4%) and the subsequent risk of virologic failure (LPV/r, 0.23%/mo; EFV, 0.15%/mo) from NEVEREST-3 data; we obtained ART costs (LPV/r, $6–$20/mo; EFV, $3–$6/mo) from published sources. We projected discounted life expectancy (LE) and lifetime costs per person. A secondary analysis used data from MONOD-ANRS-12206 in Côte d’Ivoire.

**Results:**

Continued LPV/r led to the shortest LE (18.2 years) and the highest per-person lifetime cost ($19 470). LPV/r with second-line option increased LE (19.9 years) and decreased per-person lifetime costs($16 070). Switching led to the longest LE (20.4 years) and the lowest per-person lifetime cost ($15 240); this strategy was cost saving under plausible variations in key parameters. Using MONOD-ANRS-12206 data in Côte d’Ivoire, the Switch strategy remained cost saving only compared with continued LPV/r, but the LPV/r with second-line option strategy was cost-effective compared with switching.

**Conclusion:**

For children ≥3 years old and virologically suppressed by LPV/r-based ART, preemptive switching to EFV can improve long-term clinical outcomes and be cost saving.

**Clinical Trials Registration:**

NCT01127204

## Teaser

Using the Cost-Effectiveness of Preventing AIDS Complications–Pediatric model to project clinical outcomes, costs, and cost-effectiveness, we show that preemptively switching to efavirenz in virologically suppressed children >3 years old receiving ritonavir-boosted lopinavir (LPV/r) is cost saving compared with continued LPV/r.

In 2017, there were approximately 1.8 million children aged <15 years living with human immunodeficiency virus (HIV) in sub-Saharan Africa, and 180 000 new pediatric infections each year [1]. Antiretroviral therapy (ART) dramatically reduces pediatric HIV-related mortality rates. Ritonavir-boosted lopinavir (LPV/r) has been recommended as the first ART regimen for children <3 years of age since 2013 [2–7]. Several recent studies have provided evidence on the safety and efficacy of dolutegravir (DTG). In 2018, DTG was recommended as first-line ART in children weighing ≥20 kg (approximately age 5–6 years), and it is anticipated to be recommended for younger children in the near future), as well as for second-line ART in when other regimens fail [7–9]. However, given the limited availability of DTG and other pediatric formulations in resource-limited settings, many children are still starting first-line LPV/r [4, 7].

LPV/r-based ART for young children, however, is poorly tolerated, interacts with tuberculosis medications, is costly, and may lead to long-term metabolic complications [10, 11]. Alternative approaches have been investigated, including initiating first-line LPV/r-based ART and then substituting efavirenz (EFV). In children who start LPV/r-based ART at <2 years of age and who are virologically suppressed, preemptive switching to EFV could improve tolerability, preserve LPV/r for later treatment strategies, and reduce costs. Furthermore, in children weighing >10 kg, EFV-based ART can be given once daily, potentially improving treatment adherence.

Two randomized clinical trials have compared this preemptive switch strategy to remaining on LPV/r: the NEVEREST-3 trial, conducted in South Africa, and the MONOD-ANRS-12206 trial, conducted in Burkina Faso and Côte d’Ivoire [12–14]. In both trials, children who were virologically suppressed after ≥12 months on LPV/r-based ART were randomized to switch to EFV or continue LPV/r. EFV and LPV/r showed similar short-term virologic outcomes in both studies. Both trials showed noninferiority for the outcome of “confirmed virologic failure,” defined as HIV RNA >1000 copies/mL, at 48–52 weeks after randomization. In addition, EFV led to more favorable lipid profiles and CD4 cell response in the NEVEREST-3 trial.

Whereas preemptive switching to EFV could offer advantages, the potential long-term clinical outcomes and costs of the switch strategy remain unknown. Our objective was to project the long-term clinical outcomes, costs, and cost-effectiveness of a preemptive switch to EFV in virologically suppressed children aged ≥3 years receiving LPV/r-based therapy, compared with other recommended and commonly used ART sequencing strategies [15].

## METHODS

### Analytic Overview

**Figure 1.**
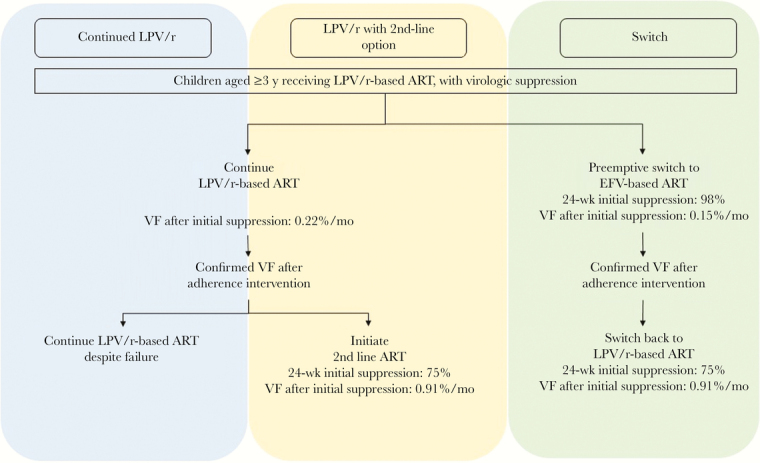
Diagram of the 3 modeled strategies. Continued LPV/r represents the current practice in most sub-Saharan African settings, where no alternative option is available after ritonavir-boosted lopinavir (LPV/r) failure. LPV/r with second-line option represents the current World Health Organization recommendations, where children failing first-line LPV/r should be switched to second-line antiretroviral therapy (ART). In the base case, we assumed this second-line option would be non-nucleoside reverse-transcriptase inhibitor based, with virologic outcomes shown here; we varied these virologic outcomes and costs widely to reflect other second-line ART options, including dolutegravir. The “switch” strategy is the strategy evaluated in the NEVEREST-3 and MONOD-ANRS-12206 trials. Abbreviations: EFV, efavirenz; VF, virologic failure.

We used the Cost-Effectiveness of Preventing AIDS Complications (CEPAC)–Pediatric model to compare 3 modeled strategies for children ≥3 years of age virologically suppressed by initial LPV/r-based therapy (Figure 1). The first is continued LPV/r during suppression; with virologic failure (defined as confirmed viremia, 2 consecutive viral load measurements >1000 copies/µL), there are no subsequent options, as is often the case in resource-limited settings [16]. The second strategy is LPV/r with second-line option; that involves continuing LPV/r but also starting a nonnucleoside reverse-transcriptase inhibitor (NNRTI)–based regimen in children with observed virologic failure, per the 2013 recommendations from the World Health Organization (WHO) [3]. The third choice is switching, that is, using the preemptive EFV switch strategy evaluated in the NEVEREST-3 and MONOD-ANRS-12206 trials, which involves switching from suppressive LPV/r to EFV. Model outcomes included short- and long-term survival, per-person HIV-related healthcare costs, and life expectancy (LE).

We calculated an incremental cost-effectiveness ratio (ICER) for each strategy, discounted at 3% per year, compared with the next least expensive alternative: the difference in lifetime costs divided by the difference in years of life saved (YLS). Following cost-effectiveness analysis convention, discounting was used to adjust future costs and LEs to their present value, reflecting common preferences for benefits that accrue in the present rather than in the future [17]. We considered interventions cost-effective if they had ICERs less than the country’s per-capita gross domestic product for 2016 ($5270 for South Africa and $1500 for Côte d’Ivoire). We recalculated virologic outcomes (initial suppression and subsequent virologic failure risks) from primary trial data as needed for the structure of the CEPAC model (Supplementary Table A). Our primary analysis modeled NEVEREST-3 data in South Africa, and we conducted a secondary analysis using MONOD-ANRS-12206 data in Côte d’Ivoire (Table 1 and Supplementary Tables A and B). In sensitivity analyses, we varied key model input parameters individually and simultaneously.

**Table 1.** Selected Model Input Parameters

Input Parameter	Value		Range for Sensitivity Analyses	Sources
Cohort characteristic	NEVEREST-3	MONOD-ANRS-12206		
Age, mean (SD), mo	46.9 (6)	26.8 (6)	36–60	[12, 13]
Male sex, %	47.5	44.3	…	
Initial CD4 cell proportion, mean (SD), %	34.7 (7)	35.2 (8)	…	
Loss to follow-up, %/mo	0.2	0.2	0–1	[18]
ART clinical inputs				
Treatment strategy				
Continued LPV/r	LPV/r + 2 NRTIs	…		[12, 19–21]
ART efficacy (RNA <1000 copies/mL at time specified), %^a^	100	…	0–99	
Time to initial suppression	Immediate	…	…	
Risk of failure after suppression, %/mo^b^	0.23	…	…	
Monthly CD4 cell gain during suppressive ART				
CD4 cell proportion, % (patients aged 0–4 y)	1st 6 mo: 0.4; after 6 mo: 0.4	…	…	
CD4 cells/µL (patients aged 5–13 y)	1st 6 mo and after 6 mo: 3.4	…	…	
LPV/r with 2nd-line option	LPV/r + 2 NRTIs	2nd line^**c**^		
ART efficacy (RNA <1000 copies/mL at time specified), %^a^	100	75	0–99	
Time to initial suppression	Immediate	24 wk	…	
Risk of failure after suppression, %/mo^b^	0.23	0.91	0.1–2	
Monthly CD4 cell gain during suppressive ART				
CD4 cell proportion, % (patients aged 0–4 y)	1st 6 mo and after 6 mo: 0.4	1st 6 mo: 2.2; after 6 mo: 0.7		
CD4 cells/µL (patients aged 5–13 y)	1st 6 mo and after 6 mo: 3.4	1st 6 mo: 67.3; after 6 mo: 3.4		
Switch	EFV + 2 NRTIs	LPV/r + 2 NRTIs^**c**^		
ART efficacy (RNA <1000 copies/mL at time specified), %^a^	98.4	75	0–99	
Time to initial suppression, wk	24	24		
Risk of failure after suppression, %/mo^b^	0.15	0.91	0.1–2	
Monthly CD4 cell gain during suppressive ART				
CD4 cell proportion, % (patients aged 0–4 y)	1st 6 mo and after 6 mo: 0.7	1st 6 mo: 1.9; after 6 mo: 0.5		
CD4 cells/µL (patients aged 5–13 y)	1st 6 mo and after 6 mo: 3.4	1st 6 mo: 67.3; after 6 mo: 3.4		
Cost inputs, $ ^**d**^				
Laboratory assay				
CD4 cell assay in South Africa	11		0.5–2-fold increase	[22] (Leigh Berrie, personal communication, 2016)
CD4 cell assay in Côte d’Ivoire	9			
Viral load assay in South Africa	21			
Viral load assay in Côte d’Ivoire	32			
ART regimen costs, $/mo (range by age/weight)^**e**^				
LPV/r (or pediatric or adult tablets)	6–13; 11–20		0.5–2-fold increase	[23, 24]
Abacavir-lamivudine (pediatric or adult tablets)	4–21			
Zidovudine-lamivudine (pediatric or adult tablets)	2–9			
EFV (pediatric or adult tablets, age ≥3 y)	3–6			

Abbreviations: ART, antiretroviral therapy; EFV, efavirenz; LPV/r, ritonavir-boosted lopinavir; NRTIs, nucleos(t)ide reverse-transcriptase inhibitors; SD, standard deviation.

^a^ART efficacy was expressed as the probability of suppressing human immunodeficiency virus RNA levels to <400 copies/mL at the time specified (immediately in children continuing with suppressive LPV/r, otherwise 24 weeks in base case analyses after initiation of ART [19–21]. Owing to small numbers of children and similar suppression rates for second-line ART in the P1060 trial (second-line nonnucleoside reverse-transcriptase inhibitor [NNRTI], n = 9; 24-week suppression, 75%; second-line protease inhibitor, n = 48; 24-week suppression, 74%), we assigned a suppression rate of 75% to both second-line regimens.

^b^The monthly risk of virologic failure for those with initially suppression on ART was calculated from the difference in suppression risks at the earliest (24 weeks) and at 48 weeks in the NEVEREST-3 and MONOD-ANRS-12206 trials and the latest observed time point in the P1060 and PENPACT-1 trials. To fit the Cost-Effectiveness of Preventing AIDS Complications model structure, these values differ slightly from those reported in published reports of these trials. (See Supplementary Table B for full details of calculations of these parameters from the NEVEREST-3 and MONOD-ANRS-12206 data.)

^c^The second-line regimen is used after observed virologic failure with the previous ART regimen. In the LPV/r with second-line option strategy, in the base case, we assumed that this second-line regimen would be an NNRTI with 2 NRTIs. With the switch strategy, we assumed it would be LPV/r with 2 NRTIs.

^d^Costs are given in 2016 US dollars.

^e^Monthly ART drug doses were calculated for children aged 0–13 years old based on the World Health Organization weight-based dosing recommendations. Daily doses were then multiplied by unit drug costs from the May 2012 Clinton Health Access Initiative antiretroviral price list to determine monthly ART costs by age and weight. All children were assumed to receive liquid/syrup drug formulations until age 5 years for LPV/r. After this age, children were assumed to transition to pediatric or adult tablet formulations based on weight-based dosing recommendations. Fixed-dose combinations were assumed to be used where available [23].The initial NRTI backbone was Zidovudine/Lamivudine and children were switched to Abacavir/Lamivudine in case of toxicity.

### Model

The CEPAC-Pediatric model is a validated patient-level, Monte Carlo, state-transition model of pediatric HIV disease in children (http://www.massgeneral.org/mpec) [15, 25, 26]. Children enter the model with CD4 cell count and age drawn from user-specified distributions and experience disease progression according to a range of literature-based parameters, including opportunistic infection (OI) and mortality risks, ART efficacy and toxicity and impact of viral load and laboratory monitoring (Supplementary Material). Effective ART decreases HIV viral load and increases CD4 cell count, leading to reduced risk of OIs and OI-related and HIV-related mortality rates. In each month, children can remain in care or be lost to follow-up, in which case they are assumed to stop ART, and return to care if a severe OI occurs. The model tracks clinical events, the amount of time spent in each health state, LE, and associated costs.

### Modeled Populations and Strategies

In our base case, all children were suppressed on LPV/r-based ART at the start of the simulation (Table 1). In the continued LPV/r arm, they continued taking LPV/r regardless of virologic failure. For LPV/r with second-line option, they switched to NNRTI-based ART if virologic failure was later observed. With the switch strategy, children were switched immediately from LPV/r to EFV-based ART at the start of the simulation; if virologic failure observed with EFV, they were switched back to an LPV/r-based regimen (Figure 1). Monitoring and switching in all strategies followed WHO guidelines [4], including CD4 cell counts every 6 months and HIV RNA tests every 12 months (Table 1) [27]. The rate of loss to follow-up was 2%/mo.

### Input Data

There were several key differences between NEVEREST-3 and MONOD-ANRS-12206 participants, including duration of suppressive LPV/r before the switch, age at switch, EFV dosing, use and dosing of previously received maternal and infant prevention of mother-to-child transmission regimens, and viral subtypes [12, 13]. Because the NEVEREST-3 trial had larger numbers and longer follow-up available, we chose to use NEVEREST-3 data for the base case analyses, including age at preemptive switch (3 years), initial suppression for each ART regimen, and subsequent monthly risk of virologic failure (Table 1, with full calculations in Supplementary Table A) [12]. The initial suppression probabilities for EFV in the switch arm were derived 24 weeks after the switch (proportion with RNA level <1000 copies/mL, 98.4%). The risk of virologic failure after this initial suppression (“late failure”) was 0.23%/mo with LPV/r and 0.15%/mo with EFV (Table 2 and Supplementary Table A). The rate of initial suppression (75%) and the late failure risk (0.91%/mo) for subsequent ART regimens initiated after failed LPV/r (for LPV/r with second-line option) or failed EFV (for the switch strategy) were derived from the P1060 and PENPACT-1 studies [19, 20].

**Table 2.** Model-Projected Base Case Clinical And Economical Outcomes In South Africa

Treatment Strategy	5-y Survival, %	15-y Survival, %	LE, y (Undiscounted)^a^	Lifetime Per-Person Costs, $ (Undiscounted)
Continued LPV/r	94.1	80.6	30.1	33 360
LPV/r with second-line option	94.2	85.4	34.7	29 400
Switch	94.2	86.1	36.4	29 690

Abbreviations: LE, life expectancy; LPV/r, ritonavir-boosted lopinavir.

^a^LEs are mean values projected by the model for a cohort of children similar to those aged 3–5 years in the NEVEREST-3 trial at the time of switch. Discounted LEs, which value life-years in the future to be worth than those in the present, are not directly comparable to clinical experience.

The NEVEREST-3 trial reported few neuropsychiatric adverse effects for EFV, so the base case rate of neurologic toxicity (leading to added costs and switching to LPV/r-based ART) was set to 0% and varied in sensitivity analyses [12]. Similarly, the NEVEREST-3 trial reported no toxicity that led to regimen change in children treated with LPV/r; as a result, the risk for toxicity with LPV/r was set to 0% [12]. In the base case, we included costs for the treatment of tuberculosis as follows. In the case of incident tuberculosis occurring with continued LPV/r or LPV/r with second-line option, we assumed a temporary switch to EFV for the duration of tuberculosis treatment; in the case of incident tuberculosis during second-line LPV/r in the switch strategy, we assumed the use of superboosted LPV/r for the 6-month duration of tuberculosis treatment.

Costs were derived from the South African Health Review and the Clinton Health Access Initiative, in 2016 US dollars (Table 1) [19–21]. The monthly ART costs were calculated according to age and weight bands; for example, the cost of ART in 2–5-year-olds was $33/mo for LPV/r-based ART and $20/mo for EFV-based ART and second-line ART after LPV/r failure (Table 1). In a secondary analysis, we derived similar data from the MONOD-ANRS-12206 trial and applied these data to a modeled population of children in Côte d’Ivoire. Complete model input data are provided in Supplementary Table B.

### Sensitivity Analyses

In univariate sensitivity analyses, we varied key model input parameters, including initial suppression (0%–100%), late failure rates (0.1%–2.0%/mo), major toxicity rates for EFV and LPV/r (1-time probability leading to regimen change, 1%–20%), and costs (0.5–2.0 times the base case) for each ART regimen; the frequency of viral load testing during first-line ART (every 3 months during EFV treatment and in all strategies); the costs of HIV care (0.5–2.0 times the base case) and HIV RNA monitoring (0.5–2.0 times the base case); age at preemptive switch (3–5 years); and loss to follow-up rate (0%–1%/mo) (Supplementary Table B).

In 2-way sensitivity analyses, we varied the probabilities of late failure with EFV in the switch strategy and of LPV/r in with second-line option and with continued LPV/r. The specific pediatric ART regimen chosen to follow virologic failure with LPV/r varies by setting; DTG may soon become more widely available for this indication [7]. To reflect a range of possible antiretroviral formulations that would be used after failed LPV/r in children treated with second-line option, we varied the virologic outcomes (initial suppression and late failure) and costs of this regimen, including values likely to reflect DTG based on adult data (initial suppression rate, 94%; late failure risk, 0.21%/mo; cost, $4/mo) [28–30].

### Secondary Analysis

In a secondary analysis, we used data from the MONOD-ANRS-12206 trial [13]. Age at the start of the simulation was 26.8 months. The rate of initial suppression with EFV was 98.1%. The late failure risk was lower for LPV/r (0.34%) than for EFV (0.72%), which was directionally different from the findings in the NEVEREST-3 trial (Table 1). For this secondary analysis, we derived pediatric HIV care costs directly from the MONOD-ANRS-12206 trial for clinical care; clinical inputs were the same as the base case analysis for children <5 years old and were derived from adults in Côte d’Ivoire for older ages [22, 23, 31, 32].

## RESULTS

### Base Case: NEVEREST-3 Data in South Africa

Projected 5-year survival rates were similar for all 3 strategies (94.1%–94.2%; Table 2). With continued LPV/r, the 15-year survival was 80.6%, and the undiscounted LE was 30.1 years (discounted LE, 18.2 years). Adding a second-line option led to substantially better outcomes, with a projected 15-year survival rate of 85.4% and an undiscounted LE of 34.7 years (discounted, 19.9 years). The switch strategy led to the highest projected 15-year survival rate (86.1%) and the longest undiscounted LE (36.4 years; discounted LE, 20.4 years).

Projected discounted lifetime HIV-related healthcare costs were lowest with the switch strategy, at $15 240 per person; this was owing to fewer person-months spent on LPV/r, a costly ART regimen (Supplementary Figure A). The next least expensive strategy was LPV/r with second-line option, at $16 070 per person. Continued LPV/r was the most expensive strategy, at $19 470 per person. Per-person discounted costs for LPV/r with second-line option and continued LPV/r began to exceed costs for the switch strategy at 1 month after model entry (Supplementary Figure B).

### Sensitivity Analyses

We identified 6 key clinical parameters which, when varied, made the LPV/r with second-line option strategy cost-effective compared with the switch strategy. For parameters affecting the switch strategy, these included the probabilities of initial suppression with EFV (threshold at which LPV/r with second-line option became cost-effective, ≤86.0%; base case value, 98.4%), late failure with EFV (threshold, ≥0.26%/mo; base case value, 0.15%), and toxicity with EFV (threshold, ≥10%/mo; base case value, 0%). For parameters affecting the LPV/r with second-line option strategy, these included the probabilities of late failure with LPV/r (threshold, ≤0.12%/mo; base case value, 0.23%) and late failure with the second-line regimen used after failure of LPV/r (threshold, ≤0.35%/mo; base case value, 0.91%) (Table 3 and Supplementary Table C).

**Table 3.** Base Case Cost-Effectiveness Outcomes Results and Selected Sensitivity Analyses in South Africa

Treatment Strategy^a^	LE, y (Discounted)^b^	Lifetime Per-Person Costs, $ (Discounted)^c^	ICER, $/YLS^d^
Base case cost-effectiveness outcomes			
Switch	20.4	15 240	…
LPV/r with 2nd-line option	19.9	16 070	More expensive, less effective
Continued LPV/r	18.2	19 470	More expensive, less effective
Key sensitivity analyses comparing switch and LPV/r with 2nd-line option strategies			
Initial suppression with EFV in switch strategy = 86%			
Switch	19.7	15 450	…
LPV/r with 2nd-line option	19.9	16 070	2880
Late failure risk with EFV in switch strategy = 0.26%/mo			
Switch	19.7	15 490	…
LPV/r with 2nd-line option	19.9	16 070	3960
Probability of major toxicity with EFV in switch strategy = 10%			
Switch	19.7	15 410	…
LPV/r with 2nd-line option	19.9	16 070	3820
Late failure risk with LPV/r in LPV/r with 2nd-line option strategy = 0.12%			
Switch	20.4	15 240	…
LPV/r with 2nd-line option	20.9	17 560	5000
Late failure risks with 2nd-line NNRTI-based ART in LPV/r with 2nd-line option = 0.35%			
Switch	20.4	15 240	…
LPV/r with 2nd-line option	20.6	16 390	5100
Loss-to-follow-up rates in all strategies = 0.8%/mo			
LPV/r with 2nd-line option	16.9	14 740	…
Switch	17.3	15 490	2150
Scenario analyses: use of DTG as 2nd-line ART			
DTG as 2nd-line ART in LPV/r with 2nd-line option only			
Switch	20.4	15 240	…
LPV/r with 2nd-line option	21.6	16 208	807
DTG as 2nd-line ART in both LPV/r with 2nd-line option and Switch			
Switch	21.9	13 672	…
LPV/r with 2nd-line option	21.6	16 208	More expensive, less effective
Secondary analysis: MONOD-ANRS-12206 trial data for children in Côte d’Ivoire			
Switch	18.1	16 750	…
LPV/r with 2nd-line option	19.4	16 800	30
Continued LPV/r	17.4	20 020	More expensive, less effective

Abbreviations: ART, antiretroviral therapy; DTG, dolutegravir; EFV, efavirenz; ICER, incremental cost-effectiveness ratio; LE, life expectancy; LPV/r, ritonavir-boosted lopinavir; NNRTI, nonnucleoside reverse-transcriptase inhibitor; YLS, years of life saved.

^a^Strategies are listed in order of increasing costs. As a result, the order of the 3 treatment strategies changes between scenarios. In comparisons of all 3 strategies, continued LPV/r remained more expensive and less effective than either of the other 2 strategies.

^b^LEs are mean values projected by the model for a cohort of children similar to those aged 3–5 years in the NEVEREST-3 trial at the time of switch. Discounted LEs, which value life-years in the future to be worth “less” than those in the present are not directly comparable to clinical experience.

^c^Costs are in 2016 US dollars. Discounting is at 3% per year.

^d^World Health Organization–CHOICE recommendations for country-specific gross domestic product–based cost-effectiveness thresholds are based primarily on cost per quality-adjusted life-year saved or cost per disability-adjusted life-year averted. Because of limited health utility weight data in children, we project non–quality-weighted LEs and thus calculate ICERs in dollars per life-year saved.

In addition to these regimen-specific parameters, results were also sensitive to variations in the monthly rate of loss to follow-up. When the loss to follow-up rate was ≥0.8%/mo (base case value, 0.2%), the switch strategy was no longer cost saving, but it remained cost-effective: the switch strategy led to longer LE and greater costs than LPV/r with second-line option, with an ICER that remained below the cost-effectiveness threshold (Supplementary Table C). In all modeled viral load monitoring scenarios, the switch strategy remained cost saving compared with the LPV/r with second-line option (Supplementary Table C).

When no second-line ART option was available in the case of failure with LPV/r, there was no LPV/r cost at which the switch strategy was not preferred over LPV/r with second-line (either cost saving or cost-effective), because LPV/r costs affect both strategies. Wide variations in all other key model input parameters, through ranges shown in Table 1, did not affect the comparison of these 2 strategies (Supplementary Table C). If the LPV/r with second-line option was assumed to be unavailable, the switch strategy remained cost saving compared with continued LPV/r, with plausible variations in all model input parameters (Supplementary Table D).

#### Scenario Analyses: Use of DTG as a Second-Line ART Option.

In a scenario in which the ART regimen after LPV/r failure in the LPV/r with second-line option strategy included DTG, using the inputs derived from adult data with the second-line option was more effective and cost-effective than the switch strategy (ICER, $807/YLS) (Table 3). However, when the regimen after EFV failure with the switch strategy was also DTG, the switch strategy led to the longest LE and lowest costs, remaining cost saving, as in the base case.

#### Multivariate Sensitivity Analyses.

**Figure 2.**
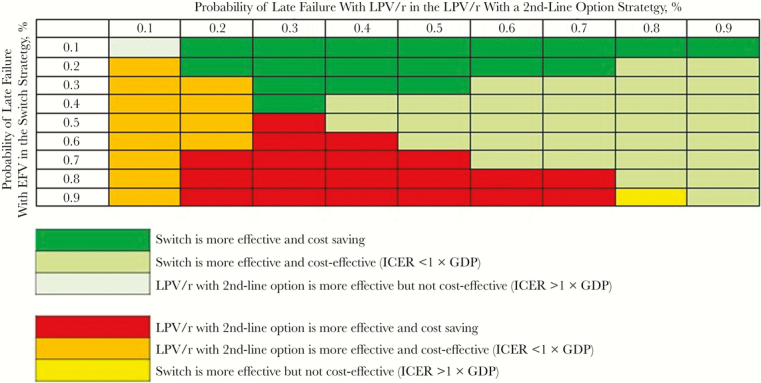
Multivariate sensitivity analyses: impact of simultaneous variation in monthly risk of late failure on preemptive switch to efavirenz (EFV) or first-line ritonavir-boosted lopinavir (LPV/r). The monthly risk of late failure of the preemptive EFV-based regimen is shown on the vertical axis; the monthly risk of late failure on the first-line LPV/r-based regimen is shown on the horizontal axis. This figure shows results comparing the switch strategy with that of LPV/r with second-line option (availability of nonnucleoside reverse-transcriptase inhibitor–based second-line antiretroviral therapy in case of failure with LPV/r). Costs and life-years are discounted at 3% per year. Following World Health Organization gross domestic product (GDP)–based guidance, cost-effectiveness results support the choice of switching in the green-shaded scenarios and the choice of LPV/r with second-line option in the red-, orange-, and yellow-shaded scenarios. Abbreviation: ICER, incremental cost-effectiveness ratio.

We simultaneously varied the risks of late failure with EFV in the switch strategy, as well as late failure with LPV/r for both LPV/r with second-line option and continued LPV/r, from 0.1%/mo to 0.9%/mo. We then compared the switch strategy with both LPV/r with second-line option (Figure 2) and continued LPV/r (Supplementary Figure C). For both comparisons, there were key combinations of high late failure risks for EFV and/or low late failure risks for LPV/r that made switching no longer the preferred strategy.

**Figure 3.**
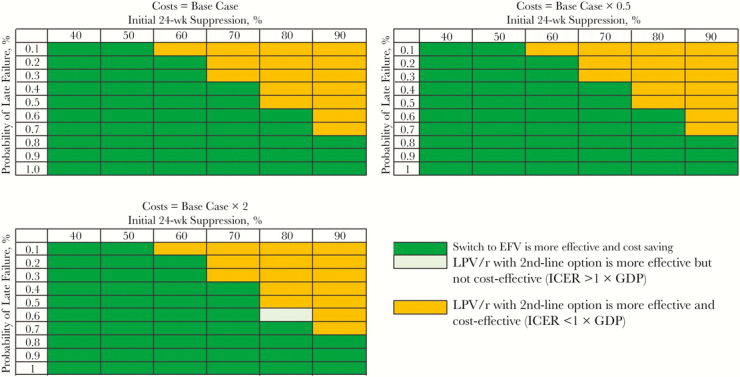
Multivariate sensitivity analyses of the impact of simultaneous variation in characteristics of the antiretroviral therapy (ART) regimen after failed ritonavir-boosted lopinavir (LPV/r) in the LPV/r with second-option strategy, including initial suppression, monthly risk of late failure, and costs. In each panel, the monthly risk of late failure of the ART regimen that would follow LPV/r failure in the LPV/r with second-line option strategy is shown on the vertical axis, and the initial 24-week suppression with that regimen is shown on the horizontal axis. The top left panel shows base case ART costs for this regimen. The top right panel shows results when the cost of this regimen is reduced by half. The bottom panel shows results when the cost is doubled. Green shading indicates scenarios in which the switch strategy is cost saving (leading to greater life expectancy and lower lifetime costs), compared with LPV/r with second-line option; pale green shading indicates that the LPV/r with second-line option strategy projects the longest life expectancy but is not cost-effective, and the switch strategy is economically preferred. Orange shading indicates scenarios wherein LPV/r with second-line option is cost-effective compared with the switch strategy. Costs and life-years are discounted at 3% per year. Abbreviations: EFV, efavirenz; GDP, gross domestic product; ICER, incremental cost-effectiveness ratio.

The clinical benefits and cost of the ART regimen that followed LPV/r failure in the LPV/r with second-line option strategy also affected the comparison of that strategy with switching (Figure 3). As the probability of initial suppression increased and/or late failure risks decreased for the second-line option, the LPV/r with second-line option strategy became economically preferred at all plausible costs.

### Secondary Analysis: MONOD-ANRS-12206 Data in Côte d’Ivoire

When using MONOD-ANRS-12206 data in Côte d’Ivoire, we projected lower LEs and higher lifetime costs for all strategies, and the policy conclusions changed (Table 3). Continued LPV/r remained the strategy with the lowest projected LE (17.4 years). LE increased with the switch strategy (18.1 years), and it increased further with the LPV/r with second-line option. The longer LE for the LPV/r with second-line option (opposite to the base case findings using NEVEREST-3 data) was due to the lower late failure rates for LPV/r (0.34%/mo) compared with EFV (0.72%/mo) reported from the MONOD-ANRS-12206 trial.

The trend in per-person lifetime costs across all 3 strategies was similar to the findings with NEVEREST-3 data: lowest with the switch strategy ($16 750), intermediate with the LPV/r with second-line option ($16 800), and highest with continued LPV/r ($20 020). Compared with the switch strategy, LPV/r with second-line option was cost-effective (ICER, $30/YLS). However, in Côte d’Ivoire, as in other West African settings, second-line ART is not widely available. Although EFV is widely available, clinicians are often reluctant to switch children with failing first-line protease inhibitors to NNRTI-based regimens, owing to concerns for lower potency and development of drug resistance, especially if poor adherence is suspected [16, 33]. Compared with Continued LPV/r, switching remained cost saving in this context.

## DISCUSSION

We used data from the NEVEREST-3 and MONOD-ANRS-12206 trials to estimate the cost-effectiveness of preemptively switching to EFV in children ≥3 years of age who are virologically suppressed with first-line LPV/r in sub-Saharan Africa. Using data from NEVEREST-3, with a larger sample size and longer follow-up time than MONOD-ANRS-12206, our primary finding was that preemptive switching to EFV was cost saving over the lifetimes of HIV-infected children, compared with continuing LPV/r.

In many African settings in 2019, ART options after virologic failure with LPV/r are limited. The comparison between LPV/r with second-line option and switching depends on the clinical characteristics of the second-line regimen used after failed LPV/r. In our base case, we assumed it would be NNRTI based, to reflect as much as possible the clinical reality in settings where the integrase inhibitors now recommended by WHO are not yet widely available [7, 34]. Clinical characteristics were derived from second-line EFV or nevirapine in the P1060 and PENPACT trials; the rate initial suppression as defined in this analysis was approximately 75% in both [19, 21, 26].

Beyond these clinical trials, few studies have actually described a “protease inhibitor then NNRTI” sequence of treatment, and in those that do, the efficacy reported is often lower than that used in our analyses, ranging from 25% to 30% [35, 36]. When we modeled these lower efficacy rates, preemptive switching to EFV remained cost saving (Figure 3). Data on regimens other than EFV after virologic failure on LPV/r are very limited for children. DTG will become more widely available in the near future; if it proves to have similar cost and clinical efficacy in children as in adults, our results suggest that LPV/r with second-line DTG may be more effective and cost-effective than preemptive switching to EFV followed by LPV/r in the case of virologic failure (Table 3).

In the absence of non-NNRTI options, many clinicians are reluctant to switch children in whom LPV/r is failing to an NNRTI-based regimen, owing to anticipated poorer efficacy, and these children often continue to receive LPV/r [16, 35]. Compared with this approach of continued LPV/r, preemptively switching to EFV remains more effective and less costly in all scenarios and settings. The 2018 WHO guidelines no longer recommend EFV as a preferred drug for children, based largely on 4 considerations: (1) rising rates of pretreatment NNRTI resistance, (2) concerns for EFV neurotoxicity, (3) new LPV/r formulations that may be better tolerated than current liquid LPV/r, and (4) the anticipated availability of DTG [7]. The impact of pretreatment NNRTI resistance was included in the estimates of both initial virologic suppression and late failure risks for first-line ART.

Despite infant exposure to prevention of mother-to-child transmission regimens and anticipated resulting drug resistance, 98% of children in both the NEVEREST-3 and MONOD-ANRS-12206 trials had virologic suppression with EFV at 6 months. In addition, although we do not directly model resistance mutations that accumulate over time, these are in part accounted for in our data inputs for initial virologic suppression and late failure risks for second-line ART. Although novel formulations of LPV/r, such as pellets [11, 37], may improve tolerability, our sensitivity analyses based on NEVEREST-3 data suggest that even with plausible lower late failure risks for LPV/r, the switch strategy remains a cost-effective or cost-saving alternative to both LPV/r strategies, while we await widespread access to DTG for children.

While robust to variations in many key model input parameters, the finding that preemptive switching to EFV was cost saving compared with continuing LPV/r with second-line option was sensitive to the probabilities of both initial suppression and later virologic failure (a lifelong risk) for each modeled ART strategy. The published MONOD-ANRS-12206 and NEVEREST-3 trial outcomes (virologic failure at 48–52 weeks, which showed noninferiority of EFV compared with LPV/r in both trials) reflect a composite of these 2 parameters. To fit the structure of the CEPAC model and make long-term projections, we separated initial suppression risk from late failure risk. Initial suppression values for EFV (98% at 6 months after preemptive switching) were nearly equivalent in the 2 trials. In contrast, late failure risks were directionally different: NEVEREST-3 estimates favored EFV (LPV/r, 0.23%/mo; EFV, 0.15%/mo), whereas MONOD-ANRS-12206 estimates favored LPV/r (LPV/r, 0.34%/mo; EFV, 0.72%/mo).

Key differences between both trial populations may explain these differences in monthly virologic failure risks after initial suppression (children in NEVEREST-3 were older, with more exposure to maternal or infant NNRTIs and longer duration of viral suppression at the time of the switch). However, because both trials concluded that the 2 strategies were clinically similar, we believe it is more likely that our calculated differences in late failure risks are due to small numbers and the resulting statistical uncertainty in long-term outcomes. The impact of small numbers was particularly pronounced in the MONOD-ANRS-12206 trial. When we calculated late failure risks using NEVEREST-3 data with longer follow-ups than used in the base case calculations (to 192 rather than 48 weeks), late failure risks favored EFV by even larger degrees (LPV/r, 0.21%/mo; EFV, 0.09%/mo) [14]. We therefore chose the NEVEREST-3 estimates for our base case and, conservatively, used the 48-week calculations.

In addition to this uncertainty around late failure risks, our study has several limitations inherent to model-based cost-effectiveness analyses. First, we extrapolate long-term outcomes from short-term data (eg, we assume that the rate of late failure with each ART regimen is constant over time and lasts for the duration of the regimen). In addition, long-term outcomes (>15 years) for children receiving ART in resource-limited settings are not yet available, so our model was calibrated to fit short-term OIs and mortality rates [15, 26], and outcomes were then projected over longer horizons. Computational requirements make probabilistic sensitivity analysis difficult in the CEPAC-Pediatric model, so we did not formally evaluate the simultaneous impact of uncertainty in all model input parameters through probabilistic sensitivity analyses. However, we evaluated uncertainty in these long-term projections through extensive sensitivity univariate and multivariate analyses, in keeping with international recommendations for modeling best practices [38, 39]. Except where noted, policy conclusions were unchanged.

In conclusion, both the NEVEREST-3 and MONOD-ANRS-12206 trials demonstrated the short-term clinical noninferiority of a preemptive switch to EFV, compared with continuing LPV/r, for children aged >3 years who are virologically suppressed after ≥12 months of LPV/r-based ART and. While we await greater access to DTG and data about its long-term effectiveness in children, a preemptive switch to EFV in children suppressed on early-initiated LPV/r should be considered in settings where second-line ART options are limited.

## Supplementary Material

ofz276_suppl_supplementary_appendix
