# Supplementary material for: Cost-Effectiveness of Preemptive Switching to Efavirenz-Based Antiretroviral Therapy for Children With Human Immunodeficiency Virus
Source: Open Forum Infect Dis. 2019 Jun 11;6(7):ofz276. doi: 10.1093/ofid/ofz276 (PMC6634435; doi:10.1093/ofid/ofz276)
Supplement: ofz276_suppl_supplementary_appendix [file ofz276_suppl_supplementary_appendix.docx]

**Cost-effectiveness of pre-emptive switching to efavirenz-based**

**antiretroviral therapy for children with HIV**

**Supplemental Appendix**

Sophie Desmonde

*et al.*

**INTRODUCTION**

This appendix is included to provide methodological details to supplement the description of the methods in the manuscript text, as well as additional model outputs and results.

**METHODS**

**Model structure**

We have previously described the structure of the CEPAC-Pediatric natural history model, reflecting HIV disease progression in the absence of ART [[1](#_ENREF_1)]. In additional manuscripts and corresponding Appendices, we provided additional detail about HIV diagnosis and treatment in the CEPAC-Pediatric model [[2-5](#_ENREF_2)]. Full details of model structure, data sources, and procedures for initiating new collaborative projects are also available on the CEPAC website, at <http://www.massgeneral.org/mpec>. Here, we describe selected aspects of model structure and data inputs that are most relevant to this analysis, in which we simulated a population of HIV-infected children initially suppressed on ritonavir-boosted lopinavir (LPV/r)-based ART.

The model tracks true CD4%/CD4 and HIV RNA level, although clinical decisions are made based on observed information, such as symptomatic illness or CD4%/CD4 or RNA levels measured according to specified laboratory monitoring strategies. In each month, children can remain in care or be lost to follow-up (LTFU). If they are LTFU, they are assumed to stop ART, and return to care if a severe opportunistic infection (OI) occurs.

For each ART regimen, we specify an “efficacy,” defined as the probability of suppressing HIV RNA to <1000 copies/µL (c/µL), and the time point by which this occurs (usually 24 weeks or 48 weeks). Each regimen also confers monthly medication costs, as well as gains in CD4% or CD4 count for children with suppressed HIV RNA. Children who initially suppress HIV RNA at 24 or 48 weeks face a monthly risk of virologic failure thereafter (“late failure”). Following virologic failure, HIV RNA slowly rises to a “set point” that is determined as a function of HIV RNA level at birth. After virologic failure, there is a 12-month delay until CD4% or CD4 count begins to decline at pre-ART rates, leading to increased monthly risks of opportunistic infections and death, until the next effective ART regimen (if available) is initiated. For children who fail ART, we assign clinical criteria (number and type of opportunistic infections), immunologic criteria (decline in CD4% or CD4 count), or virologic criteria (increase in HIV RNA) by which this failure is detected, as well as the type and frequency of monitoring and confirmatory testing. After observed failure, patients can be switched to the next available line of therapy. We also incorporate a reduction in mortality and opportunistic infection risks for children on ART, independent of CD4 level and HIV RNA suppression, as observed in adults; this parameter was used for model calibration (see below) [[6](#_ENREF_6)].

For each simulated infant, the model tracks clinical events; the amount of time spent in each health state, and associated costs. After an individually simulated patient has died, the next infant enters the model. Large cohorts (1 million-10 million total patients) are simulated in order to generate stable model outcomes. Once the entire cohort has been simulated, summary statistics are tallied, including number and type of clinical events, the proportion alive each month, health care costs in each month, and life expectancy (mean for the cohort).

**Model input data**

Data used as input parameters for the CEPAC-Pediatric model are described in the main manuscript, with selected data inputs in Manuscript Table 1 and complete inputs in Appendix Table B, below.

The base case analyses used data from the NEVEREST-3 trial [[7](#_ENREF_7)]. The NEVEREST-3 trial included a small proportion of children aged >5 years; for this analysis, we derived inputs from trial data only from children aged 3-5 years at randomization. For scenario analyses, we also derived efficacy and late failure derived from the MONOD ANRS 12206 trial [[8](#_ENREF_8)]. In contrast to NEVEREST-3, MONOD ANRS 12206 found higher monthly rates of “late failure” on EFV compared to LPV/r.

Data for 2^nd^-line ART regimens were from P1060 and PENPACT-1 trials, limiting data to children <3 years of age at trial entry treated with nevirapine or LPV/r [[9](#_ENREF_9), [10](#_ENREF_10)]

**Opportunistic infections in children <5 years of age included WHO stage 3 events, WHO stage 4 events, and tuberculosis, derived from the IeDEA cohort (Appendix Table B) [**[**11**](#_ENREF_11)**]. In the base-case, we included costs for the treatment of tuberculosis (TB) as follows: in case of incident TB occurring on LPV/r in *Continued LPV/r* or *LPV/r with second-line option*, we assumed a temporary switch to EFV for the duration of TB treatment; in case of incident TB on second-line LPV/r in *Switch*, we assumed super-boosted LPV/r for the 6-month duration of TB treatment. As a conservative assumption with regard to the benefit of *Switch*, we assumed no change in clinical efficacy, only changes to regimen costs. Because including the costs associated with ART regimen changes during TB treatment changed the base case results only minimally (lifetime costs differed by <$15/person, and policy conclusions were not changed), we did not include these cost modifications in all of the sensitivity analyses.**

**Model calibration**

*Natural history model calibration*

We first calibrated our model to fit observed data for children in the absence of ART; this is described in detail in a previous manuscript [[1](#_ENREF_1)]. In brief, we first internally validated the CEPAC-Pediatric model to assess the accuracy of model structure. We did this by using input data from the IeDEA East Africa region, and ensuring that model-projected survival and OI rates matched the data used as inputs [[11](#_ENREF_11)]. We next calibrated the model to pooled survival rates from >1,300 children with *in utero* or intrapartum HIV infection in 12 PMTCT studies, pooled by the UNAIDS Child Survival Group [[12](#_ENREF_12), [13](#_ENREF_13)]. This involved increasing the rates of HIV-related mortality to account for survivor biases and differences in access to clinical care for children in the IeDEA cohort compared to children in the pooled UNAIDS analysis [[1](#_ENREF_1)].

*On-ART model calibration*

We next calibrated our model to fit observed data on mortality rates, OI rates, and rates of switching from first-line to 2^nd^-line ART. This work is described in the Appendix of a previous publication and summarized here [[2](#_ENREF_2)].

In adults, a reduction in risks of OIs and death has been reported for patients on ART, regardless of whether ART is suppressive and in addition to the reduction in risk conferred by improvements in CD4 count alone, although data remain equivocal [[6](#_ENREF_6)]. We used adult data for this "ART-associated CD4-independent reduction in OI and mortality risk" for simulated subjects after the age of 13. In the absence of data on a similar ART effect in children, we used this parameter to calibrate the model for children <13 years of age to fit observed OI and mortality rates in the P1060 trial [[10](#_ENREF_10)]. We first attempted to match the mortality rates observed in the P1060 trial (3.29/100PY). The best fit to the P1060-observed mortality rate was found with relative reductions of 85-95% in the relative risk of mortality that occurs outside of the 30 days immediately following an opportunistic infection. Holding the relative risk reduction in mortality at 90% (the midpoint of this range), we next compared model-generated OI rates using these multipliers to P1060-observed rates of WHO stage 3, WHO stage 4, and tuberculosis events. These were found to match most closely when the relative reduction in opportunistic infection risk was 85%. Finally, we compared the life expectancies projected to result from relative reductions in OI risk of 85-95% and relative reductions in mortality risk of 90-95%. There are no empiric data to inform the life expectancy of HIV-infected African children treated with modern ART regimens. Based on projected results for adults, we felt that life expectancies in the 27-28-year range for children surviving to 12 months of age before ART, as observed with relative risk reductions of 85% and 90%, were most reasonable [[14](#_ENREF_14)]. We thus selected relative risk reductions of 85% for mortality and 90% for opportunistic infection as our final calibrated parameters. In sensitivity analyses, we varied these values widely from 0-100%.

*Rates of switch from 1^st^-line to 2^nd^-line ART*

Monitoring and switching followed WHO guidelines [[15](#_ENREF_15)]. Monitoring included CD4 tests every 6 months and HIV RNA tests at 6 and 12 months after regimen change, then 12-monthly thereafter [[16](#_ENREF_16)]. **We evaluated the impact of quarterly VL monitoring in two sensitivity analyses.**

**1. First, we simulated more frequent VL testing in the children who pre-emptively switched to EFV: quarterly VL during the time they were on EFV. This scenario paralleled the trial protocol (quarterly VL only while on EFV in the Switch strategy), which was based on a concern that virologic failure might occur more frequently in the Switch strategy, leading to the potential risk for accumulated drug resistance if EFV was continued after failure. As this was not observed in both the NEVEREST-3 and MONOD trials, we felt it was unlikely that the Switch strategy would be implemented with quarterly VL monitoring, and we therefore conducted this as a sensitivity analysis rather than in the base case analysis.**

**2. Second, we compared the three modelled ART strategies under the assumption that quarterly VL testing would be offered to children on first-line ART in all three strategies. It seems unlikely, when practiced in routine care, that additional VL would be only conducted in the sub-group of children with pre-emptive switch (especially if suppressed) while others would receive annual or biannual monitoring.**

For those pre-emptively switching to EFV and those initiating 2^nd^-line ART, detection and confirmation of ART failure was only possible after more than 24 weeks on ART. Failure was modeled using the criteria below:

- Virologic failure: Observed RNA >1,000 copies/mL, confirmed by a second RNA test at least 1 month after the first.
- Immunologic failure: Observed CD4% <10% (for children <5 years old) or CD4 count <100/µL (for children >5 years old), confirmed by a RNA test at least 1 month after the first.
- Clinical failure: Observed three new or recurring WHO Stage 3/4 or TB events, confirmed by an RNA test at least 1 month after the clinical event.

**Sensitivity analyses**

The model accounts for first-order uncertainty (between-patient variability) through the microsimulation of large cohorts of patients. Following the guidance of the International Society for Pharmacoeconomics and Outcomes Research (ISPOR), we examine the impact of second-order uncertainty (uncertainty in data parameters and assumptions) through wide-ranging univariate and multivariate sensitivity analyses on all model input parameters and assumptions [[3](#_ENREF_3), [17](#_ENREF_17)].

The main manuscript contains key univariate and multivariate sensitivity analyses, identifying the thresholds for key parameters at which clinical or cost-effectiveness results change. Appendix Tables C and D provide additional detail for these sensitivity analyses, demonstrating wider ranges through which inputs were varied in order to more clearly show trends in cost and survival outcomes. This appendix also includes additional multivariate sensitivity analyses that were not included in the main manuscript: low relative risk reduction for OIs and mortality on ART combined with low probability of developing an opportunistic infection; all sensitivity analyses are in the context of South Africa (OI rates and healthcare costs).

**RESULTS**

In the main manuscript, we show the results of the base case analysis, as well as sensitivity analyses in which the results differed from the base case. All other sensitivity analyses did not lead to changes in policy conclusions, except where noted in the manuscript. Appendix Tables C and D show results for selected values of the parameters that were varied.

**Appendix Table A - Monthly probability of late virologic failure (“late failure”) in the NEVEREST-3 and MONOD-ANRS-12206 trials.**

|  | **NEVEREST-3** | | **MONOD-ANRS-12206** | |
| --- | --- | --- | --- | --- |
|  | **LPV/r** | **EFV** | **LPV/r** | **EFV** |
| Number treated | 121 | 121 | 54 | 52 |
| Time point of initial suppression (T1) , months | 0 | 6 | 0 | 6 |
| Number of children 2xVL* > 1000c/µl at T1 | 0 | 2 | 0 | 1 |
| Number of children 2xVL* > 1000c/µl at 48 weeks | 3 | 3 | 2 | 3 |
| **Initial suppression rate (at T1)** | **100.0%** | **98.3%** | **100.0%** | **98.1%** |
| Suppression rate at 48 weeks (T2) | 97.5% | 97.5% | 96.3% | 94.2% |
| **Monthly probability of late failure^†^** | **0.23%** | **0.15%** | **0.34%** | **0.72%** |
| ***Additional inputs for the NEVEREST-3 trial at 96 and 192 weeks.*** | | |  |  |
| Number of children 2xVL *> 1000c/ml at 96weeks | 7 | 3 |  |  |
| Suppression rate at 96 weeks (T2) | 94.2% | 97.5% |  |  |
| **Monthly probability of late failure^†^** | **0.27%** | **0.05%** |  |  |
| Number of children 2xVL* > 1000c/ml at 192 weeks | 11 | 6 |  |  |
| Suppression rate at 192 weeks (T2) | 90.9% | 95.0% |  |  |
| **Monthly probability of late failure^†^** | **0.21%** | **0.08%** |  |  |

* VL: viral load; virologic failure was defined in the base case as two consecutive viral load measurements >1000 copies.

**^†^**To calculate a probability of late failure, we used the following formula:$1-\left( \frac{\% suppressed at T1}{\% suppressed at T2} \right)^{\left( \frac{1}{T1+T2}\times\frac{52}{12} \right)}$. We used the 48-week time point in our base case for consistency with the MONOD trial, where longer-term follow-up was not available. Results using the 96- and 192-week time points can be found in the multivariate sensitivity analyses.

**Appendix Table B. Model input parameters (including those shown in Manuscript Table 1)**

| **I. Cohort characteristics** | **Value** | | **Sources** |
| --- | --- | --- | --- |
|  | **Neverest-3** | **Monod** |  |
| Age, m (SD) | 46.9 (6) | 26.8 (6) | [[7](#_ENREF_7), [8](#_ENREF_8)] |
|  | (sensitivity analyses: 36-60 months) | |  |
| Percent male | 47.5% | 44.3% |  |
| Initial CD4% (SD) | 34.7% (7%) | 35.2% (8%) |  |
| Initial viral load (VL) | <500 copies/mL | |  |
| Lost to follow-up (LTFU), monthly | 0.2% | | [[18](#_ENREF_18)] |
|  | (sensitivity analyses: 0-1%) | |  |
| **II. Natural History Clinical Inputs** | **South Africa** | **Cote d’Ivoire** |  |
| **Rate of CD4%/ CD4 decline** | Monthly Risk (%) | | [[11](#_ENREF_11), [19](#_ENREF_19)] |
| <3 months of age (CD4%) | 4.0% | |  |
| 3-59 months of age (CD4%) | 0.5% | |  |
| ≥60 months of age (CD4 cells/µL, range by HIV RNA) | 3.0-6.4 | |  |
| **Risk of clinical events (range by CD4%)** | Monthly Risk (%) | |  |
| 6-59 months of age |  |  |  |
| WHO Stage 3 event (except tuberculosis) | 3.3-11.6 | | [[11](#_ENREF_11), [19](#_ENREF_19)] |
| WHO Stage 4 event (except tuberculosis) | 1.4-6.4 | |  |
| Tuberculosis (any body site) | 0.8-3.8 | |  |
| ≥60 months of age |  |  |  |
| Mild fungal infection | 1.8-3.1 | 0.7-9.4 | [[6](#_ENREF_6), [11](#_ENREF_11), [19-22](#_ENREF_19)] |
| Visceral bacterial infection | 0.04-0.71 | 0.1-1.7 |  |
| WHO Stage 3 or 4 visceral disease | 0.03-1.4 | 0.02-2.9 |  |
| WHO Stage 3 or 4 mucocutaneous disease | 0.03-2.3 | 0.01-2.7 |  |
| Other WHO Stage 3 or 4 disease | 0.02-0.73 | 0.8-3.7 |  |
| Other severe disease | 0.19-1.7 | 0.3-2.0 |  |
| Other mild disease | 2.4 | 0.7-4.0 |  |
| Tuberculosis (any body site) | 0.03-1.7 | 0.05-0.6 |  |
| **Risk of death within 30 days of clinical event** | 30-day risk (%) | |  |
| 0-59 months of age |  |  | [[6](#_ENREF_6), [11](#_ENREF_11), [19-22](#_ENREF_19)] |
| After WHO Stage 3 or 4 event | 13.5 | |  |
| After TB event | 11.1 | |  |
| ≥60 months of age |  |  |  |
| Mild fungal infection | 0.5 | 6.5-22.2 |  |
| Visceral bacterial infection | 2.9 | 3.2-16.7 |  |
| WHO Stage 3 or 4 visceral disease | 9.2 | 6.6-12.3 |  |
| WHO Stage 3 or 4 mucocutaneous disease | 2.4 | 0.0 |  |
| Other WHO Stage 3 or 4 disease | 20.0 | 6.6-12.3 |  |
| Other severe disease | 6.7 | 3.2-16.7 |  |
| Other mild disease | 0.4 | 0.0 |  |
| Tuberculosis (any body site) | 1.8 | 6.5-22.2 |  |
| **Risk of non AIDS-related mortality (range by age in yearly intervals, sex, country)** | Monthly risk (%) | |  |
| <12 months of age | 0.41-0.49 | | [[23](#_ENREF_23)] |
| 12-60 months of age | 0.05 | |  |
| 5-13 years of age | 0.01 | |  |
| 13-18 years of age | 0.01 | |  |
| >18 years of age | 0.01-0.10 | |  |

**Appendix Table B, continued**

| **III. ART Clinical Inputs for Neverest-3** | **Values** | | **Source** |
| --- | --- | --- | --- |
| *1)     Continued LPV/r* | *LPV + 2NRTIs* | | [[7](#_ENREF_7), [9](#_ENREF_9), [10](#_ENREF_10), [24](#_ENREF_24)] |
| ART efficacy (RNA <1000c/ml at time specified)^a^ | 100% | |  |
|  | (sensitivity analyses: 0-99%) | |  |
| Time to initial suppression | Immediate | |  |
| Monthly risk of failure after suppression^b^ | 0.23% | |  |
| Monthly CD4%/CD4 gain on suppressive ART (1^st^ 6m, after 6m) | | |  |
| Ages 0-4 years, CD4% | 0.4%, 0.4% | |  |
| Ages 5-13 years, CD4 cells/µl | 3.4, 3.4 | |  |
| Monitoring | CD4 every 6m, VL every 12m | |  |
| *2)     LPV/r with second-line option* | *LPV + 2NRTIs* | *Second-line* **^c^** |  |
| ART efficacy (RNA <1000c/ml at time specified)^a^ | 100% | 75% |  |
|  | (sensitivity analyses: 0-99%) | |  |
| Time to initial suppression | Immediate | 24 weeks |  |
| Monthly risk of failure after suppression^b^ | 0.23% | 0.91% |  |
|  | (sensitivity analyses: 0.1-2%) | |  |
| Monthly CD4%/CD4 gain on suppressive ART (1^st^ 6m, after 6m) | | |  |
| Ages 0-4 years, CD4% | 0.4%, 0.4% | 2.2%, 0.7% |  |
| Ages 5-13 years, CD4 cells/µl | 3.4, 3.4 | 67.3, 3.4 |  |
| Monitoring | CD4 every 6m, VL every 12m | CD4 every 6m |  |
| *3)     Switch* | *EFV + 2NRTIs* | *LPV + 2NRTIs* |  |
| ART efficacy (RNA <1000c/ml at time specified)^a^ | 98.4% | 75% |  |
|  | (sensitivity analyses: 0-99%) | |  |
| Time to initial suppression | 24 weeks | 24 weeks |  |
| Monthly risk of failure after suppression^b^ | 0.15% | 0.91% |  |
|  | (sensitivity analyses: 0.1-2%) | |  |
| CD4%/CD4 gain on suppressive ART (1^st^ 6m, after 6m) | | |  |
| Ages 0-4 years, CD4% | 0.7%, 0.7% | 1.9%, 0.5% |  |
| Ages 5-13 years, CD4 cells/µl | 3.4, 3.4 | 67.3, 3.4 |  |
| Monitoring | CD4 every 6m, VL every 12m | CD4 every 6m |  |

**Appendix Table B, continued.**

| **IV. ART Clinical Inputs for Monod** | **Values** | | **Source** |
| --- | --- | --- | --- |
| *1)     Continued LPV/r* | *LPV + 2NRTIs* | | [[8-10](#_ENREF_8), [24](#_ENREF_24)] |
| ART efficacy (RNA <1000c/ml at time specified)^a^ | 100% | |  |
|  | (sensitivity analyses: 0-99%) | |  |
| Time to initial suppression | Immediate | |  |
| Monthly risk of failure after suppression^b^ | 0.34% | |  |
| CD4%/CD4 gain on suppressive ART (1^st^ 6m, after 6m) | | |  |
| Ages 0-4 years, CD4% | 0.4%, 0.4% | |  |
| Ages 5-13 years, CD4 cells/µl | 3.4, 3.4 | |  |
| Monitoring | CD4 every 6m, VL every 12m | |  |
| *2)     LPV/r with second-line option* | *LPV + 2NRTIs* | *Second-line* **^c^** |  |
| ART efficacy (RNA <1000c/ml at time specified)^a^ | 100% | 75% |  |
|  | (sensitivity analyses: 0-99%) | |  |
| Time to initial suppression | Immediate | 24 weeks |  |
| Monthly risk of failure after suppression^b^ | 0.34% | 0.91% |  |
|  | (sensitivity analyses: 0.1-2%) | |  |
| CD4%/CD4 gain on suppressive ART (1^st^ 6m, after 6m) | | |  |
| Ages 0-4 years, CD4% | 0.4%, 0.4% | 2.2%, 0.7% |  |
| Ages 5-13 years, CD4 cells/µl | 3.4, 3.4 | 67.3, 3.4 |  |
| Monitoring | CD4 every 6m, VL every 12m | CD4 every 6m |  |
| *3)     Switch* | *EFV + 2NRTIs* | *LPV + 2NRTIs* |  |
| ART efficacy (RNA <1000c/ml at time specified)^a^ | 98.1% | 75% |  |
|  | (sensitivity analyses: 0-99%) | |  |
| Time to initial suppression | 24 weeks | 24 weeks |  |
| Monthly risk of failure after suppression^b^ | 0.72% | 0.91% |  |
|  | (sensitivity analyses: 0.1-2%) | |  |
| CD4%/CD4 gain on suppressive ART (1^st^ 6m, after 6m) | | |  |
| Ages 0-4 years, CD4% | 0.7%, 0.7% | 1.9%, 0.5% |  |
| Ages 5-13 years, CD4 cells/µl | 3.4, 3.4 | 67.3, 3.4 |  |
| Monitoring | CD4 every 6m, VL every 12m | CD4 every 6m |  |

**Appendix Table B, continued.**

| **V. ART Clinical Inputs – both trials** | **Values** | **Sources** |
| --- | --- | --- |
| **Reduction in event risk for patients on ART** | **Relative risk reduction (%)^d^** |  |
| Risk of opportunistic infection (age 0-13) | 85 | [[6](#_ENREF_6)] |
| Risk of opportunistic infection (age 13+) | 32 |  |
| Mortality risk (age 0-13) | 90 |  |
| Mortality risk (age 13+, range by CD4) | 55-96 |  |
| **VI. Cost Inputs** | **Cost (2016 USD)** |  |
| **Opportunistic infection care (per event; range by type of OI)** | |  |
| South Africa (<60 months of age) | $800 -1500 | [[25](#_ENREF_25)] |
| Côte d’Ivoire (<60 months of age) | $125-445 | [[26](#_ENREF_26)] |
| South Africa (≥60 months of age) | $200-650 | [[19](#_ENREF_19), [20](#_ENREF_20)] |
| Côte d’Ivoire (≥60 months of age) | $60-420 | [[20](#_ENREF_20)] |
| **Routine care (per month, range by CD4)** |  |  |
| South Africa (<60 months of age) | $17-129 | [[27](#_ENREF_27), [28](#_ENREF_28)] |
| Côte d’Ivoire (<60 months of age) | $20-170 | [[26](#_ENREF_26)] |
| South Africa (≥60 months of age) | $17-129 | [[19](#_ENREF_19), [20](#_ENREF_20)] |
| Côte d’Ivoire (≥60 months of age) | $30-40 | [[19](#_ENREF_19), [20](#_ENREF_20)] |
| **Care in the last month of life** |  |  |
| South Africa | $500 | [[27](#_ENREF_27), [28](#_ENREF_28)] |
| Côte d’Ivoire | $65 | [[26](#_ENREF_26), [28](#_ENREF_28)] |
| **Laboratory assays** |  |  |
| CD4 Assay in South Africa | $11 | [[26](#_ENREF_26), [29](#_ENREF_29)] |
| CD4 Assay in Cote d’Ivoire | $9 |  |
| VL Assay in South Africa | $21 |  |
| VL Assay in Cote d’Ivoire | $32 |  |
| **Antiretroviral regimen costs (per month, range by age/weight)^e^** | |  |
| Lopinavir/ritonavir (liquid: age <3 years or pediatric or adult tablets) | $6-13; $11-20 | [[30](#_ENREF_30), [31](#_ENREF_31)] |
|  | (Sensitivity analysis: ↑0.5-2x) |  |
| Abacavir/lamivudine (pediatric or adult tablets) | $4-21 |  |
| Zidovudine/lamivudine (pediatric or adult tablets) | $2-9 |  |
| Efavirenz (pediatric or adult tablets, age ≥3 years) | $3-6 |  |

**WHO**: World Health Organization. **TB**: tuberculosis. **LPV/r:** lopinavir/ritonavir. **EFV:** efavirenz

a. ART efficacy: probability of suppressing HIV RNA to <400 copies/mL by 24 weeks (in base case analysis) after initiation of ART. Due to small numbers of children and similar suppression rates on second-line ART in the P1060 trial (second-line NNRTI: n=9, 24-week suppression=75%; second-line PI: n=48, 24-week suppression=74%), we assigned a suppression rate of 75% to both second-line regimens.

b. The monthly risk of virologic failure for those who initially suppress on ART was calculated from the difference in suppression risks at the earliest (24 weeks) and 48 weeks in the NEVEREST-3 and MONOD ANRS 12206 trials and the latest observed time point in the P1060 and PENPACT-1 trials.

c. Second-line regimen is used after observed virologic failure on the previous ART regimen. In *LPV/r with second-line option*, in the base case, we assumed this second-line regimen would be a non-nucleoside reverse transcriptase inhibitor (NNRTI) with 2 NRTIs. In *Switch*, we assumed it would be LPV/r with 2 NRTIs.

d. Compared to children not on ART with similar CD4 (see text).

e. Monthly ART drug doses were calculated for children ages 0-13 years old based on the WHO weight-based dosing recommendations. Daily doses were then multiplied by unit drug costs from the May 2012 Clinton Health Access Initiative (CHAI) ARV price list to determine monthly ART costs by age and weight. All children were assumed to receive liquid/syrup drug formulations until age 3 years for lopinavir/ritonavir (5 years in sensitivity analyses), and until age 6 months for all other medications, for which dispersible tablets are available. After these ages, children were assumed to transition to pediatric or adult tablet formulations based on weight-based dosing recommendations. Fixed dose combinations were assumed to be used where available [[31](#_ENREF_31)].

**Appendix Table C. Sensitivity analysis results comparing *Switch* to *LPV/r with second-line option*, South Africa**

| **Treatment strategy ^a^** | **Life expectancy^b^ (years, discounted)** | **Lifetime costs (USD per person, discounted)** | **Incremental cost-effectiveness ratio^c^ ($/YLS)** |
| --- | --- | --- | --- |
| **Alternative probabilities of initial suppression on EFV in *Switch* (base case = 98%)** | | | |
| **Initial suppression on EFV in *Switch* = 99%** | |  |  |
| *Switch* | 20.5 | $15,200 |  |
| *LPV/r with second-line option* | 19.9 | $16,080 | More expensive, less effective |
| **Initial suppression on EFV in *Switch* = 86%** | |  |  |
| *Switch* | 19.7 | $15,450 |  |
| *LPV/r with second-line option* | 19.9 | $16,080 | $2,880 |
| **Initial suppression on EFV in *Switch* = 85%** | |  |  |
| *Switch* | 19.6 | $15,480 |  |
| *LPV/r with second-line option* | 19.9 | $16,080 | $2,100 |
| **Late failure risks on EFV in *Switch* (base case = 0.15%/month)** | | | |
| **Late failure risk on EFV in *Switch* = 0.25%/ month** | | | |
| *Switch* | 19.8 | $15,510 |  |
| *LPV/r with second-line option* | 19.9 | $16,080 | $9,100 |
| **Late failure risk on EFV in *Switch* = 0.26%/ month** | | | |
| *Switch* | 19.7 | $15,490 |  |
| *LPV/r with second-line option* | 19.9 | $16,080 | $3,960 |
| **Late failure risks on LPV/r in *LPV/r with second line option* (base case = 0.23%/month)** | | | |
| **Late failure risk on 1st-line LPV/r = 0.12%/ month** | |  |  |
| *Switch* | 20.4 | $15,220 |  |
| *LPV/r with second-line option* | 20.9 | $17,560 | $5,000 |
| **Late failure risk on 1st-line LPV/r = 0.13%/ month** | |  |  |
| *Switch* | 20.4 | $15,220 |  |
| *LPV/r with second-line option* | 20.8 | $17,520 | $6,030 |
| **Late failure risk on 1st-line LPV/r = 0.2%/ month** | |  |  |
| *Switch* | 20.4 | $15,220 |  |
| *LPV/r with second-line option* | 20.1 | $16,140 | More expensive, less effective |

**Appendix Table C. Sensitivity analysis results comparing *Switch* to *LPV/r with second-line option*, South Africa – continued**

| **Treatment strategy^a^** | **Life expectancy^b^ (years, discounted)** | **Lifetime costs (USD per person, discounted)** | **Incremental cost-effectiveness ratio^c^ ($/YLS)** |
| --- | --- | --- | --- |
| **Late failure risks on 2nd line ART in *LPV/r with second line option* (base case = 0.91%/month)** | | | |
| **Late failure on 2^nd^-line ART in *LPV/r with second line option* = 0.35%/month** | | | |
| *Switch* | 20.4 | $15,220 |  |
| *LPV/r with second-line option* | 20.6 | $16,390 | $5,100 |
| **Late failure on 2^nd^-line ART in *LPV/r with second line option* = 0.40%/month** | | | |
| *Switch* | 20.4 | $15,220 |  |
| *LPV/r with second-line option* | 20.5 | $16,320 | $10,990 |
| **Probabilities of initial suppression on 2nd line ART in *LPV/r with second line option* (base case = 75%)** | | | |
| **Initial suppression on 2^nd^-line ART in *LPV/r with second line option* = 99%** | | | |
| *Switch* | 20.4 | $15,220 |  |
| *LPV/r with second-line option* | 20.5 | $16,330 | $16,170 |
| **Initial suppression on 2^nd^-line ART in *LPV/r with second line option* = 90%** | | | |
| *Switch* | 20.4 | $15,220 |  |
| *LPV/r with second-line option* | 20.3 | $16,270 | More expensive, less effective |
| **Probability of major toxicity on EFV in *Switch* (base case = 0%)** | | |  |
| **Probability of major toxicity on EFV = 7%** | |  |  |
| *Switch* | 20.0 | $15,340 |  |
| *LPV/r with second-line option* | 19.9 | $16,080 | More expensive, less effective |
| **Probability of major toxicity on EFV = 9%** | |  |  |
| *Switch* | 19.8 | $15,410 |  |
| *LPV/r with second-line option* | 19.9 | $16,080 | $12,630 |
| **Probability of major toxicity on EFV = 10%** | |  |  |
| *Switch* | 19.7 | $15,410 |  |
| *LPV/r with second-line option* | 19.9 | $16,080 | $3,820 |
| **Monthly loss-to-follow-up rates in all strategies (base case = 0.2%)** | | | |
| **Loss to follow-up = 0%** |  |  |  |
| *Switch* | 22.3 | $14,300 |  |
| *LPV/r with second-line option* | 21.6 | $16,740 | More expensive, less effective |
| **Loss to follow-up = 0.4%** |  |  |  |
| *Switch* | 19.1 | $15,490 |  |
| *LPV/r with second-line option* | 18.7 | $15,580 | More expensive, less effective |
| **Loss to follow-up = 0.8%** |  |  |  |
| *LPV/r with second-line option* | 16.9 | $14,740 |  |
| *Switch* | 17.3 | $15,490 | $2,150 |
| **Loss to follow-up = 1%** |  |  |  |
| *LPV/r with second-line option* | 16.3 | $14,440 |  |
| *Switch* | 16.6 | $15,380 | $3,030 |

**Appendix Table C. Sensitivity analysis results comparing *Switch* to *LPV/r with second-line option*, South Africa - continued**

| **Treatment strategy^a^** | **Life expectancy^b^ (years, discounted)** | **Lifetime costs (USD per person, discounted)** | **Incremental cost-effectiveness ratio^c^ ($/YLS)** |
| --- | --- | --- | --- |
| **Costs of LPV/r (in all strategies)** |  |  |  |
| **Costs x 0.5** |  |  |  |
| *Switch* | 20.4 | $14,000 |  |
| *LPV/r with second-line option* | 19.9 | $14,130 | More expensive, less effective |
| **Costs x 0.75** |  |  |  |
| *Switch* | 20.4 | $14,620 |  |
| *LPV/r with second-line option* | 19.9 | $15,140 | More expensive, less effective |
| **Costs x 2** |  |  |  |
| *Switch* | 20.4 | $17,620 |  |
| *LPV/r with second-line option* | 19.9 | $20,050 | More expensive, less effective |
| **Costs of VL assay (in all strategies)** |  |  |  |
| **Costs x 0.5** |  |  |  |
| *Switch* | 20.4 | $14,080 |  |
| *LPV/r with second-line option* | 19.9 | $14,920 | More expensive, less effective |
| **Costs x 2** |  |  |  |
| *Switch* | 20.4 | $17,500 |  |
| *LPV/r with second-line option* | 19.9 | $18,470 | More expensive, less effective |
| **Costs of 2nd line ART regimen in *LPV/r with second line* option** | | |  |
| **Costs x 0.5** |  |  |  |
| *Switch* | 20.4 | $15,220 |  |
| *LPV/r with second-line option* | 19.9 | $15,640 | More expensive, less effective |
| **Costs x 2** |  |  |  |
| *Switch* | 20.4 | $15,220 |  |
| *LPV/r with second-line option* | 19.9 | $16,970 | More expensive, less effective |
| **Costs x 5** |  |  |  |
| *Switch* | 20.4 | $15,220 |  |
| *LPV/r with second-line option* | 19.9 | $19,550 | More expensive, less effective |
| **CD4 independent impact of ART on OI and mortality risks (base case 85/90% relative risk reduction) (in all strategies)** | | | |
| **No impact on OI or mortality risks** |  |  |  |
| *Switch* | 16.4 | $13,010 |  |
| *LPV/r with second-line option* | 15.9 | $13,690 | More expensive, less effective |
| **50% relative risk reduction of OI and mortality** | |  |  |
| *Switch* | 18.6 | $14,200 |  |
| *LPV/r with second-line option* | 18.1 | $15,030 | More expensive, less effective |

**Appendix Table C. Sensitivity analysis results comparing *Switch* to *LPV/r with second-line option*, South Africa – continued**

| **Treatment strategy^a^** | **Life expectancy^b^ (years, discounted)** | **Lifetime costs (USD per person, discounted)** | **Incremental cost-effectiveness ratio^c^ ($/YLS)** |
| --- | --- | --- | --- |
| **Age at model entry (base case = 46.9 months)** | | | |
| **36 months** |  |  |  |
| *Switch* | 20.4 | $14,000 |  |
| *LPV/r with second-line option* | 19.9 | $14,130 | More expensive, less effective |
| **60 months** |  |  |  |
| *Switch* | 20.5 | $15,290 |  |
| *LPV/r with second-line option* | 20.1 | $16,170 | More expensive, less effective |
| **VL monitoring frequency (base case = every 12 months)** | | | |
| **Quarterly VL monitoring in *Switch* only** | | | |
| *Switch* | 20.4 | $15,993 |  |
| *LPV/r with second-line option* | 19.9 | $16,080 | More expensive, less effective |
| **Quarterly VL monitoring in all strategies** | | | |
| *Switch* | 20.4 | $15,993 |  |
| *LPV/r with second-line option* | 19.9 | $16,827 | More expensive, less effective |
| **Probability of major toxicity on LPV/r in *LPV/r with second-line option* (base case = 0%)** | | | |
| **Probability of major toxicity on LPV/r = 5%** | | | |
| *Switch* | 20.4 | $15,993 |  |
| *LPV/r with second-line option* | 19.6 | $16,036 | More expensive, less effective |

Costs are in 2016 USD. Discounting is at 3% per year. ART: antiretroviral therapy; EFV: efavirenz; LPV/r: lopinavir/ritonavir; ICER: incremental cost-effectiveness ratio; VL: viral load.

a: Strategies are listed in order of increasing costs. As a result, the order of the three treatment strategies changes between scenarios.

b: Life expectancies are mean values projected by the model for a cohort of children similar to those aged 3-5 years in the NEVEREST-3 trial at time of switch. Discounted life expectancies, which value life-years in the future to be worth ‘less’ than life-years in the present, are not directly comparable to clinical experience.

c: WHO-CHOICE recommendations for country specific gross domestic product (GDP)-based cost-effectiveness thresholds are based primarily on cost per quality-adjusted life-year saved or cost per disability-adjusted life-year averted. Because of limited health utility weight data in children, we project non quality-weighted life expectancy, and thus calculate ICERs in dollars per life-year saved.

**Appendix Table D - Sensitivity analysis results comparing *Switch* (pre-emptive switching to EFV) to *Continued LPV/r* (continued LPV/r with no option available in case of failure) in South Africa (using NEVEREST-3 data)**

| **Treatment strategy^a^** | **Life expectancy^b^ (years, discounted)** | **Lifetime costs (USD per person, discounted)** | **Incremental cost-effectiveness ratio^c^ ($/YLS)** | |
| --- | --- | --- | --- | --- |
| **Probabilities of initial suppression on EFV in *Switch* (base case = 98%)** | | | | |
| **Initial suppression on EFV in *Switch* = 70%** | | | | |
| *Switch* | | 18.7 | $15,800 |  |
| *Continued LPV/r* | | 18.2 | $19,470 | More expensive, less effective |
| **Initial suppression on EFV in *Switch* = 55%** | | | | |
| *Switch* | | 17.7 | $16,060 |  |
| *Continued LPV/r* | | 18.2 | $19,470 | $7,280 |
| **Initial suppression on EFV in *Switch* = 53%** | | |  |  |
| *Switch* | | 17.6 | $16,180 |  |
| *Continued LPV* | | 18.2 | $19,470 | $5,460 |
| **Initial suppression on EFV in *Switch* = 52%** | | |  |  |
| *Switch* | | 17.5 | $16,140 |  |
| *Continued LPV* | | 18.2 | $19,470 | $4,800 |
| **Late failure risks on EFV in *Switch* (base case = 0.15%)** | | | | |
| **Late failure risk on EFV in *Switch* = 0.5%/month** | | | | |
| *Switch* | | 18.8 | $15,960 |  |
| *Continued LPV/r* | | 18.2 | $19,470 | More expensive, less effective |
| **Late failure risk on EFV in *Switch* = 1%/ month** | | | | |
| *Switch* | | 17.4 | $16,340 |  |
| *Continued LPV* | | 18.2 | $19,470 | $4,270 |
| **Late failure risks on LPV/r in *Continued LPV/r* (base case = 0.23%/month)** | | | | |
| **Late failure risk on 1st-line LPV/r = 0.1%/ month** | | |  |  |
| *Switch* | | 20.4 | $15,220 |  |
| *Continued LPV/r* | | 19.3 | $20,160 | More expensive, less effective |
| **Late failure risk on 1st-line LPV/r = 1%/ month** | | |  |  |
| *Switch* | | 20.4 | $15,220 |  |
| *Continued LPV/r* | | 14.7 | $17,280 | More expensive, less effective |
| **Probability of major toxicity on EFV in *Switch* (base case = 0%)** | | | | |
| **Probability of major toxicity on EFV = 5%** | | |  |  |
| *Switch* | | 20.1 | $15,310 |  |
| *Continued LPV/r* | | 18.2 | $19,470 | More expensive, less effective |
| **Probability of major toxicity on EFV = 9%** | | | | |
| *Switch* | | 19.8 | $15,410 |  |
| *Continued LPV/r* | | 18.2 | $19,470 | More expensive, less effective |

**Appendix Table D. Sensitivity analysis results comparing *Switch* to *Continued* *LPV/r*, South Africa – continued**

| **Treatment strategy ^a^** | **Life expectancy^b^ (years, discounted)** | **Lifetime costs (USD per person, discounted)** | **Incremental cost-effectiveness ratio^c^ ($/YLS)** |
| --- | --- | --- | --- |
| **Lost-to-follow-up rates (base case = 0.2%/month) (all strategies)** | | | |
| **Loss to follow-up = 0%** | | | |
| *Switch* | 22.3 | $14,300 |  |
| *Continued LPV/r* | 20.3 | $20,880 | More expensive, less effective |
| **Loss to follow-up =1%** | | | |
| *Switch* | 16.6 | $15,380 |  |
| *Continued LPV* | 14.4 | $16,730 | More expensive, less effective |
| **Alternative costs of LPV/r (all strategies)** | | | |
| **Costs x 0.5** | | | |
| *Switch* | 20.4 | $14,000 |  |
| *Continued LPV/r* | 18.2 | $16,450 | More expensive, less effective |
| **Costs x 2** |  |  |  |
| *Switch* | 20.4 | $17,620 |  |
| *Continued LPV/r* | 18.2 | $25,480 | More expensive, less effective |
| **Alternative costs of VL assay (all strategies)** | | | |
| **Costs x 0.5** | | | |
| *Switch* | 20.4 | $14,080 |  |
| *Continued LPV/r* | 18.2 | $17,130 | More expensive, less effective |
| **Costs x 2** |  |  |  |
| *Switch* | 20.4 | $17,500 |  |
| *Continued LPV/r* | 18.2 | $24,110 | More expensive, less effective |
| **CD4 independent impact of ART on OI and mortality risks (base case 85/90% relative risk reduction) (all strategies)** | | | |
| **No impact on OI or mortality risks** | | | |
| *Switch* | 16.4 | $13,010 |  |
| *Continued LPV/r* | 14.6 | $16,410 | More expensive, less effective |
| **50% relative risk reduction of OI and mortality** | | | |
| *Switch* | 18.6 | $14,200 |  |
| *Continued LPV/r* | 16.5 | $18,060 | More expensive, less effective |
| **Age at model entry** | | | |
| **36 months** | | | |
| *Switch* | 20.4 | $14,000 |  |
| *Continued LPV/r* | 18.2 | $16,450 | More expensive, less effective |
| **60 months** | | | |
| *Switch* | 20.5 | $15,290 |  |
| *Continued LPV/r* | 18.2 | $19,470 | More expensive, less effective |

**Appendix Table D. Sensitivity analysis results comparing *Switch* to *Continued* *LPV/r*, South Africa – continued**

| **Treatment strategy ^a^** | **Life expectancy^b^ (years, discounted)** | **Lifetime costs (USD per person, discounted)** | **Incremental cost-effectiveness ratio^c^ ($/YLS)** |
| --- | --- | --- | --- |
| **Probability of major toxicity on LPV/r in *LPV/r with second-line option* (base case = 0%)** | | | |
| **Probability of major toxicity on LPV/r = 5%** | | | |
| *Switch* | 20.4 | $15,993 |  |
| *Continued LPV/r* | 17.5 | $18,769 | More expensive, less effective |

Costs are in 2016 USD. Discounting is at 3% per year. ART: antiretroviral therapy; EFV: efavirenz; LPV/r: lopinavir/ritonavir; ICER: incremental cost-effectiveness ratio. a: Strategies are listed in order of increasing costs. As a result, the order of the three treatment strategies changes between scenarios. b: Life expectancies are mean values projected by the model for a cohort of children similar to those aged 3-5 years in the NEVEREST-3 trial at time of switch. Discounted life expectancies, which value life-years in the future to be worth ‘less’ than life-years in the present, are not directly comparable to clinical experience. c: WHO-CHOICE recommendations for country specific gross domestic product (GDP)-based cost-effectiveness thresholds are based primarily on cost per quality-adjusted life-year saved or cost per disability-adjusted life-year averted. Because of limited health utility weight data in children, we project non quality-weighted life expectancy, and thus calculate ICERs in dollars per life-year saved.

**
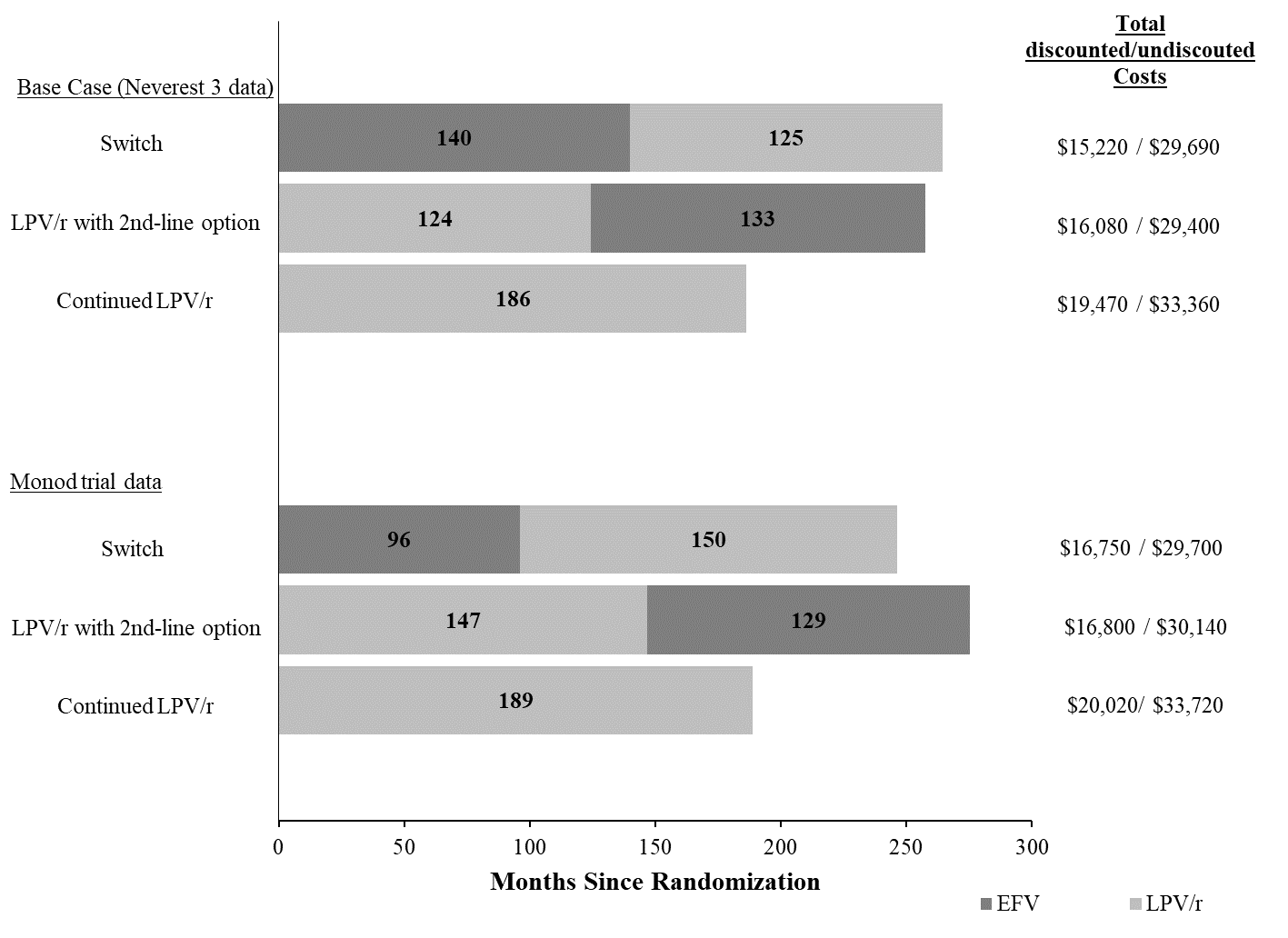
**

**Figure A – Duration on each modeled antiretroviral therapy regimen and impact on total lifetime costs.** Months spent on EFV-based ART (dark grey bars) and LPV-based ART (light grey bars) are shown, in discounted months, for base case and MONOD trial data described in the text in a cohort of children aged 3-5 years on suppressive LPV/r-based ART. Total per-person, discounted lifetime costs are shown at the right of the bars for each treatment strategy.

ART: antiretroviral therapy; EFV: efavirenz; LPV/r: lopinavir/ritonavir.


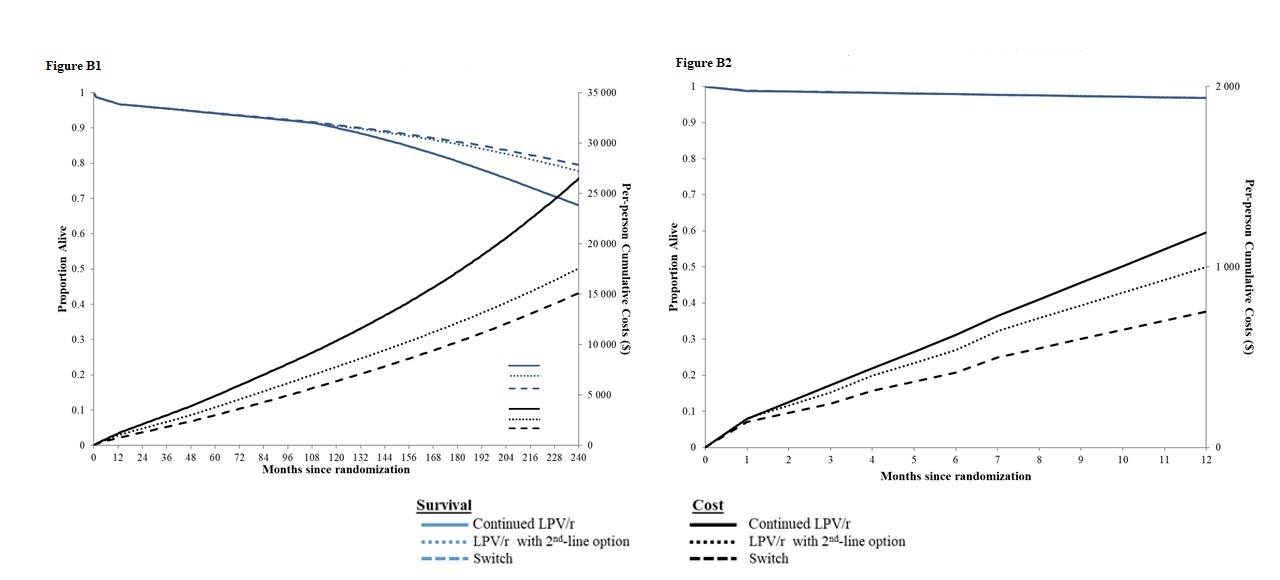


**Figure B – Projected survival and discounted costs with alternate treatment strategies**. Results are shown for the base case analysis in South Africa, using NEVEREST-3 clinical data. The proportion of patients alive is on the left-hand vertical axis (blue lines) and the per-person cumulative costs are on the right-hand vertical axis (black lines). Survival and per-person discounted costs are projected over 20 years since randomization, shown on the horizontal axis. The *Continued LPV/r* strategy is represented by solid lines, the *LPV/r with second-line option* strategy by dotted lines and the *Switch* strategy by dashed lines. Figure B1 shows results projected over 20 years. Figure B2 shows results over the first 12 months.


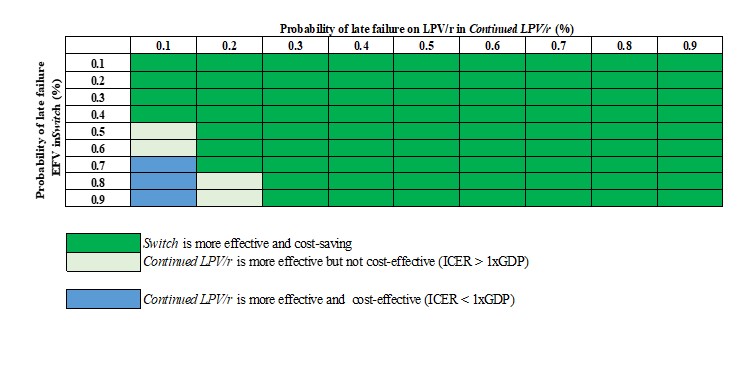


**Figure C – Multivariate sensitivity analyses: impact of simultaneous variation in monthly risk of late failure on pre-emptive switch to EFV or first-line LPV/r.** The monthly risk of late failure of the pre-emptive EFV-based regimen is shown on the vertical axis; the monthly risk of late failure on the first-line LPV/r-based regimen is shown on the horizontal axis. This figure shows results comparing *Switch* to *Continued LPV/r* with no second line options. Costs and life-years are discounted at 3% per year. Following WHO GDP-based guidance, cost-effectiveness results support the choice of *Switch* in the green-shaded scenarios and choice of *Continued LPV/r* in the blue shaded scenarios.

ART: antiretroviral therapy; GDP: gross domestic product; ICER: incremental cost-effectiveness ratio; LPV/r: lopinavir/ritonavir; EFV: efavirenz, YLS: years of life saved

**REFERENCES**

1. Ciaranello A, Morris BJ, Walensky RP, et al. Validation and calibration of a computer simulation model of paediatric HIV infection. PLosOne **2013**; 8(12).

2. Ciaranello AL, Doherty K, Penazzato M, et al. Cost-effectiveness of first-line antiretroviral therapy for HIV-infected African children less than 3 years of age. Aids **2015**; 29(10): 1247-59.

3. Dunning L, Francke JA, Mallampati D, et al. The value of confirmatory testing in early infant HIV diagnosis programmes in South Africa: A cost-effectiveness analysis. PLoS Med **2017**; 14(11): e1002446.

4. Frank SC, Cohn J, Dunning L, et al. Clinical effect and cost-effectiveness of incorporation of point-of-care assays into early infant HIV diagnosis programmes in Zimbabwe: a modelling study. Lancet HIV **2019**; 6(3): e182-e90.

5. Francke JA, Penazzato M, Hou T, et al. Clinical Impact and Cost-effectiveness of Diagnosing HIV Infection During Early Infancy in South Africa: Test Timing and Frequency. J Infect Dis **2016**; 214(9): 1319-28.

6. Losina E, Yazdanpanah Y, Deuffic-Burban S, et al. The independent effect of highly active antiretroviral therapy on severe opportunistic disease incidence and mortality in HIV-infected adults in Cote d'Ivoire. Antiviral therapy **2007**; 12(4): 543-51.

7. Coovadia A, Abrams EJ, Strehlau R, et al. Efavirenz-based antiretroviral therapy among nevirapine-exposed HIV-infected children in South Africa: a randomized clinical trial. Jama **2015**; 314(17): 1808-17.

8. Dahourou DL, Amorissani-Folquet M, Malateste K, et al. Efavirenz-based simplification after successful early lopinavir-boosted-ritonavir-based therapy in HIV-infected children in Burkina Faso and Cote d'Ivoire: the MONOD ANRS 12206 non-inferiority randomised trial. BMC Med **2017**; 15(1): 85.

9. Babiker A, Darbyshire J, Pezzotti P, et al. Changes over calendar time in the risk of specific first AIDS-defining events following HIV seroconversion, adjusting for competing risks. International Journal of Epidemiology **2002**; 31(5): 951-8.

10. Palumbo P, Lindsey JC, Hughes MD, et al. Antiretroviral treatment for children with peripartum nevirapine exposure. N Engl J Med **2010**; 363(16): 1510-20.

11. Ciaranello AL, Lu Z, Ayaya S, et al. Incidence of WHO Stage 3 and 4 events, tuberculosis, and mortality in untreated, HIV-infected children enrolling in care before 1 year of age: an IeDEA (International Epidemiologic Databases to Evaluate AIDS) East Africa regional analysis. Pediatr Infect Dis J **2014**; 33(6): 623-9.

12. Becquet R, Marston M, Dabis F, et al. Children who acquire HIV infection perinatally are at higher risk of early death than those acquiring infection through breastmilk: a meta-analysis. PloS one **2012**; 7(2): e28510.

13. Marston M, Becquet R, Zaba B, et al. Net survival of perinatally and postnatally HIV-infected children: a pooled analysis of individual data from sub-Saharan Africa. Int J Epidemiol **2011**; 40(2): 385-96.

14. Johnson LF, Mossong J, Dorrington RE, et al. Life expectancies of South African adults starting antiretroviral treatment: collaborative analysis of cohort studies. PLoS Med **2013**; 10(4): e1001418.

15. World Health Organisation. Consolidated guidelines on the use of antiretroviral drugs for treating and preventing HIV infections. Available at: <http://apps.who.int/iris/bitstream/10665/208825/1/9789241549684_eng.pdf?ua=1>. Accessed March 6th.

16. South African Department of Health. The South African antiretroviral treatment guidelines. South Africa: South African Department of Health, **2013**.

17. Briggs AH, Weinstein MC, Fenwick EA, Karnon J, Sculpher MJ, Paltiel AD. Model parameter estimation and uncertainty: a report of the ISPOR-SMDM Modeling Good Research Practices Task Force--6. Value Health **2012**; 15(6): 835-42.

18. Fox MP, Rosen S. Systematic review of retention of pediatric patients on HIV treatment in low and middle-income countries 2008-2013. Aids **2015**; 29(4): 493-502.

19. Holmes CB, Wood R, Badri M, et al. CD4 decline and incidence of opportunistic infections in Cape Town, South Africa: implications for prophylaxis and treatment. Journal of acquired immune deficiency syndromes **2006**; 42(4): 464-9.

20. Anglaret X, Chene G, Attia A, et al. Early chemoprophylaxis with trimethoprim-sulphamethoxazole for HIV-1-infected adults in Abidjan, Cote d'Ivoire: a randomised trial. Cotrimo-CI Study Group. Lancet **1999**; 353(9163): 1463-8.

21. Seyler C, Messou E, Gabillard D, Inwoley A, Alioum A, Anglaret X. Morbidity before and after HAART initiation in Sub-Saharan African HIV-infected adults: a recurrent event analysis. AIDS Res Hum Retroviruses **2007**; 23(11): 1338-47.

22. Strategies for Management of Antiretroviral Therapy Study G, El-Sadr WM, Lundgren J, et al. CD4+ count-guided interruption of antiretroviral treatment. N Engl J Med **2006**; 355(22): 2283-96.

23. United Nations. World Population Prospects: The 2008 Revision. Available at: <http://www.un.org/esa/population/publications/wpp2008/wpp2008_highlights.pdf>. Accessed 7 July.

24. Violari A, Lindsey JC, Hughes MD, et al. Nevirapine versus ritonavir-boosted lopinavir for HIV-infected children. N Engl J Med **2012**; 366(25): 2380-9.

25. Thomas L. Costing of HIV/AIDS services at a tertiary level hospital in Gauteng Province. Available at: <http://wiredspace.wits.ac.za/handle/10539/2008>. Accessed March 18th.

26. Desmonde S, Avit D, Petit J, et al. Costs of care of HIV-infected children initiating Lopinavir/Ritonavir-based antiretroviral therapy before the age of two in Cote d'Ivoire. PloS one **2016**; 11(12): e0166466.

27. Ciaranello AL, Chang Y, Margulis AV, et al. Effectiveness of pediatric antiretroviral therapy in resource-limited settings: a systematic review and meta-analysis. Clin Infect Dis **2009**; 49(12): 1915-27.

28. Yazdanpanah Y, Losina E, Anglaret X, et al. Clinical impact and cost-effectiveness of co-trimoxazole prophylaxis in patients with HIV/AIDS in Côte d'Ivoire: a trial-based analysis. Aids **2005**; 19(12): 1299-308.

29. Dr. Leigh Berrie at National Health Laboratory Services South Africa. Personal communication. **2016**.

30. Initiative CHA. Antiretroviral (ARV) ceiling prist list. .

31. Doherty K, Essajee S, Penazzato M, Holmes C, Resch S, Ciaranello A. Estimating age-based antiretroviral therapy costs for HIV-infected children in resource-limited settings based on World Health Organization weight-based dosing recommendations. BMC health services research **2014**; 14: 201.
